# Improving Stem Cell Clinical Trial Design and Conduct: Development of a Quality Assessment Tool for Stem Cell Clinical Trials

**DOI:** 10.1155/2020/8836372

**Published:** 2020-11-07

**Authors:** Yixuan Li, Li Xie, Jie Wang, Jinghang He, Ying Qian, Weituo Zhang, Zhaohui Wu, Biyun Qian

**Affiliations:** ^1^Hongqiao International Institute of Medicine, Shanghai Tongren Hospital and Faculty of Public Health, Shanghai Jiao Tong University School of Medicine, Shanghai, China; ^2^Clinical Research Institute, Shanghai Jiao Tong University School of Medicine, Shanghai, China; ^3^China Medicinal Biotech Association, Beijing 100022, China; ^4^Shanghai Clinical Research Promotion and Development Center, Shanghai Hospital Development Center, Shanghai 200041, China

## Abstract

**Background:**

Clinical trials are at the cornerstone of evidence-based stem cell therapies, but the quality assessment for designing and conduct these sometimes-complex studies are scarce of evidence. This study is aimed at developing a handy quality assessment tool for stem cell clinical trials, enhancing capacity of the self-regulate overall quality, and participating protection.

**Methods:**

The framework of quality assessment tool was based on the PQRS (progress-quality-regulation-scientific) quality assessment tool, and detailed quality indicators were developed by leader group discussion, expert consulting, and literature review. Stem cell clinical trials were retrieved from the International Clinical Trials Registry Platform, and corresponding quality indicators were assessed and extracted. The validity and feasibility of conceptual quality assessment tool were further evaluated by using structural equation modeling.

**Results:**

The quality assessment tool for stem cell clinical trials contains four critical quality attributes, including participant protection, scientific value, quality control, and stem cell products, and 9 observed quality indicators. From 11 primary clinical trial registries in the International Clinical Trials Registry Platform, 9410 stem cell trial registrations were identified, and 1036 studies were eligible for publications and protocols screening. After reviewed full text, 37 studies were included in the validity and feasibility evaluation: 32 studies were completed, and 3 studies terminated early. Most of the studies (83.79%) were in the early phase, and 63.16% of the studies were investigator-initiated trial. To further tested for validity, the critical quality attributes and quality indicators (QIs) between expertise further validated by the SEM method, which showed a good fit for the model (chi − square = 26.008; *P* = 0.353; TLI = 0.967; CFI = 0.978; RMSEA = 0.048). Compared with exploratory trials, evaluating using the quality assessment tool, confirmatory trials performed similarly in participant protection, scientific value, and quality control, but lower in stem cell products.

**Conclusions:**

The results of critical quality attributes and quality indicators between expertise and confirmatory validation analysis are basically consistent, indicating the feasibility and validity of applying this quality assessment tool for overall quality evaluation of stem cell trials.

## 1. Background

The promising benefit of stem cell therapy to unmet medical need is widely recognized [1, 2], leading to a growing number of stem cell clinical trials worldwide in recent years. Clinical practitioners and patients are expecting more and rigorous evidence to support the clinical use of stem cell therapy in treating disease without effective treatment, such as Alzheimer's disease and cardiovascular diseases.

Randomized clinical trials are the widely accepted gold standard for assessing interventions to improve health and wellbeing. Clinical trials addressing important health care questions are often multicenter, large-scale, prospective studies, which are costly, complicated, and involved multidisciplinary professional roles [3]. When properly performed, the clinical trial is one of the best methods for evaluating the efficacy of one or more interventions. However, many challenges may emerge in the aspects of study design, start-up, recruitment, data quality, and reporting of results. Inefficiencies or inadequacies in the design and conduct of clinical trials may result in wasted research resources, harm to the participant, or in extreme circumstances, not completing or answering the research question. Previously studies estimated that 85% of researches were inefficiencies or inadequacies in the conduct [4]. Several risk factors may result in inefficiencies or inadequacies of trial conducting, such as studies that designed without a systematic review of the available evidence [5] or that addressed scientific questions of less importance to patients [5] or that failed to take adequate measures to reduce potential bias [6] or that inadequately reported results of studies [7]. Since December 8, 2019, China and the rest of the world have experienced an epidemic disease of a new beta-coronavirus known as coronavirus disease 2019 (COVID-19) [8]. In response to the COVID-19 outbreak, China has more than 202 running or pending clinical trials on potential treatments for COVID-19, including stem cell therapy [9]. However, scientists warn that only carefully conducted trials could determine which measures are effective [10]. Improving the design and conduct of stem cell clinical trials is a crucial strategy for reducing research waste and enhancing protection of participants.

Over the past decade, the quality of clinical trials has shifted from reactive after-the-fact activity to one focused on the crucial work of generating evidence from well-designed trials [11]. Several attempts on the development of risk-adaptive-based clinical trial quality control tools had been made and proved to be efficient with less administrative cost [12, 13]. However, in particular concerns of stem cell therapies in human bodies and distinct characteristics of living stem cell, no studies were found for quality assessment tools designed for stem cell clinical trial context. The main goal of this study is to develop a handy quality assessment tool for stem cell trials to enhance the capacity of self-regulate overall quality and participant protection, as well as inform clinical practice and future research in using stem cell therapies tackling unmet medical needs.

## 2. Methods

### 2.1. Development of Preliminary Stem Cell Quality Indicators

The basic framework of preliminary stem cell quality indicators was based on general clinical trial quality assessment framework, PQRS (progress-quality-regulation-scientific value) quality assessment tool, applied by Clinical Research Institute at Shanghai Jiao Tong University (JCRI) to evaluate clinical trials. The PQRS quality assessment tool was developed according to widely accepted guidelines, including International Conference on Harmonization Good Clinical Practice E6(R1) (ICH E6(R1)) [14], World Health Organization Good Clinical Practice (WHO GCP) [15], SPIRIT 2013 Statement [16], and CONSORT 2010 statement [17]. JCRI is aimed at supporting high-quality, efficient, effective, and sustainable clinical trial research. Since 2017, JCRI has developed a comprehensive quality assessment tool for clinical trials to support clinical trials with high-quality trial conduct and successfully applied on quality assessment of 306 studies.

**Figure 1:**
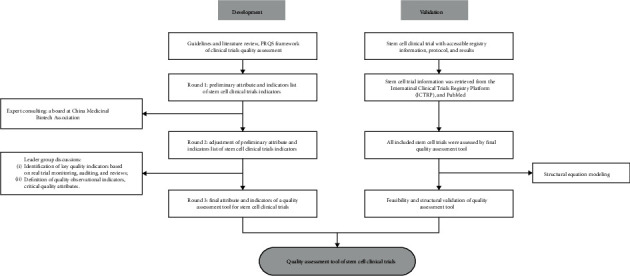
Flowchart of development and validation of quality assessment tool for stem cell clinical trials.

The specific risk and quality concerns in terms of stem cell trials were developed by leader group discussion, expert consulting method, literature review of regulatory guidelines, and peer-reviewed publications. The leader group that developed the stem cell trial QIs included diverse roles in clinical trials: those were one principle investigator who holds a Ph.D. in epidemiology and run a lab, two statisticians, a project manager, a data manager, two clinical research coordinators and two clinical research associates, all staffs holding at least master's degrees and GCP certifications, and experienced in clinical research or pharmacies for at least one year. In addition, a board at China Medicinal Biotech Association (CMBA) advised on the quality attributes and indicators (Figure 1).

### 2.2. Inclusion Criteria of Included Stem Cell Clinical Trials for Validation

Stem cell trial information was retrieved from the International Clinical Trials Registry Platform (ICTRP) at World Health Organization by using keywords of “stem cell” or “progenitor cell” or “precursor cell” or “stromal cell” or “pluripotent cell” or “bone marrow.” We included stem cell clinical trials that are (1) on human subject, (2) with the use of stem cells or progenitor cells, (3) primary purpose of treatment, (4) with at least one result publication, and (5) with protocol attachment or published protocol. Animal or vitro studies, observational studies, investigate supportive care surrounding stem cell therapy, studies without protocols were excluded. Two reviewers (YX Li and L Xie) review the abstract independently to screen for eligibility. If two reviewers made different conclusions, a third reviewer (BY Qian) would review the abstract and make the final decisions. Studies included were reviewed all and full accessible texts, including all published clinical results and attachment, to abstract data based on data-abstraction SOPs.

### 2.3. Data Collection of Measurement of Quality Indicators

Data were collected based on the review of published protocols, supplementary materials describing designs, methods, and results in publications, and information registered on the trial registry websites. QIs are proportions, which dominators are expected components and numerators observed components. All text reviewers were trained in the study protocols and standard procedures reviewing the literature.

### 2.4. Statistical Methods

Descriptive of basic characteristics of included stem cell clinical trials was presented by mean ± standard deviation or percentage, as appropriate. A structural equation model (SEM) was conducted, focusing on the pathway from observational QIs to the quality of a stem cell trial. The overall quality of stem cell trial was the second-order latent variables, which indicators were latent variable quality attributes. Model fit was evaluated using the Tucker-Lewis index (TLI), comparative fit index (CFI), and the root mean square error of approximation (RMSEA). TLI and CFI values ≥ 0.90 and RMSEA ≤ 0.08 reflect acceptable fit [18]. Analyses were carried out using the R software, “lavaan” package, and “semPlot” package (R version 3.5.3). Two-sided *P* values of less than 0.05 were considered to indicate statistical significance.

## 3. Results

### 3.1. The Framework of Quality Indicators for Stem Cell Trial

**Figure 2:**
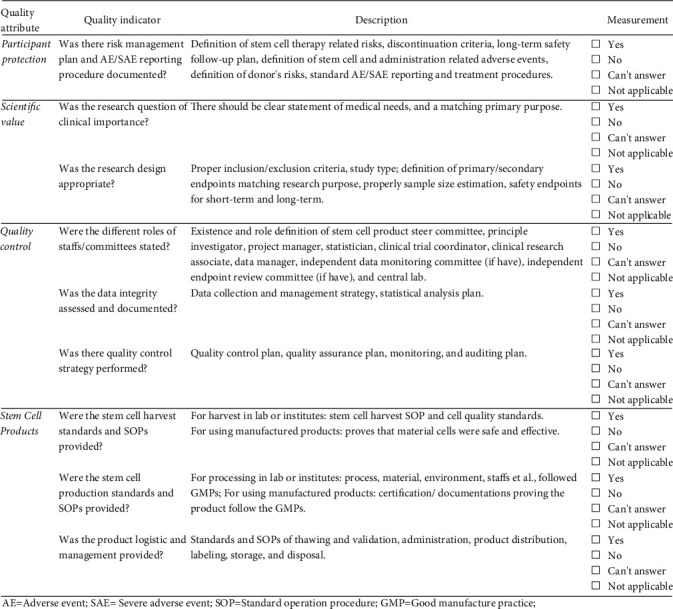
Framework of attribute and indicators of quality assessment tool for stem cell clinical trials. AE: adverse event; SAE: severe adverse event; SOP: standard operation procedure; GMP: Good Manufacture Practice.

Through literature review, leader group discussion, and expert consulting, the conceptual quality indicators (QIs) for stem cell trial contain four themes: four general quality attributes including *participant protection*, *scientific value*, *quality control* [19, 20], plus another specific component, namely, *stem cell products*. As shown in Figure 2, twelve indicators reflect the five quality themes and are developed to cover the design and conduct stage of a clinical trial. For quantitative analysis, each QI was scored based on the proportion of components found in research files.

### 3.2. Characteristics of Included Stem Cell Clinical Trial

**Figure 3:**
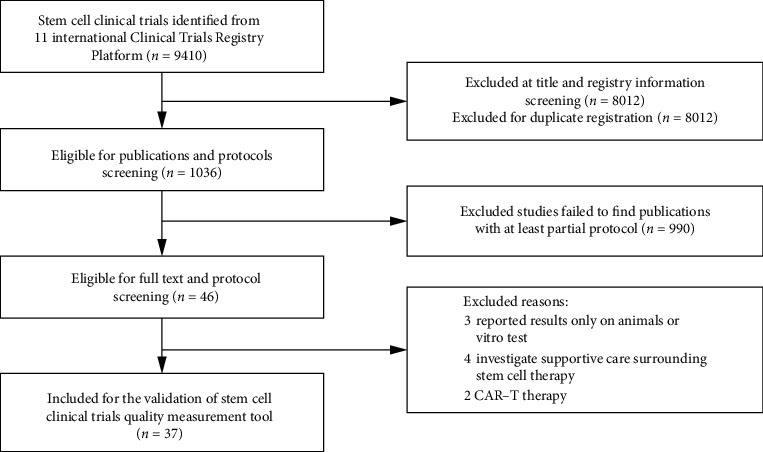
Searching and screening of stem cell clinical trials eligible for validation of quality assessment tool.

From 11 primary registries in the International Clinical Trials Registry Platform, 9410 stem cell clinical trials were identified (Figure 3). After title and registry information screening and deletion of duplication, 1036 studies were eligible for publications and protocols screening. Nine hundred and ninety studies were excluded for abstract review as no publications or results published with no protocols identified at PubMed. Two reviewers reviewed the abstracts independently and agreed on the exclusion of nine studies, including two CAR-T research, three animal or laboratory reports, and four studies observed stem cells as research outcomes. Finally, 37 studies were included for validation of quality indicators for stem cell clinical trials: 32 (86.49%) studies were completed, and 3 (8.10%) studies terminated early (Table 1). Majority of the studies (83.79%) were in the early phase of clinical trial (phase 1, phase 2, and phase 1/2), and 62.16% of the studies were initiated by investigators. Somatic stem cells (91.89%) were the most common cell type used in clinical trials; only three studies (8.11%) used pluripotent stem cells.

### 3.3. Validation of Quality Assessment Tool of Stem Cell Clinical Trial

**Figure 4:**
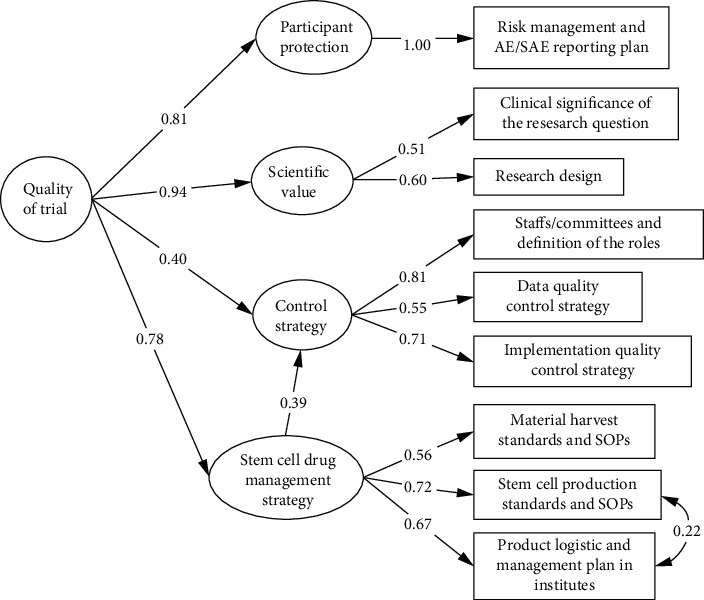
Structural equation modeling (SEM) of key indicators to the quality of stem cell trials.

The preliminary quality indicators of stem cell clinical trial quality assessment tools were further evaluated the construct validity of by using the structural equation modeling (SEM) (Figure 4). The critical quality attributes and quality indicators between expertise and confirmatory validation analysis are basically consistent, and the SEM model was adequately powered and had satisfactory goodness of fit indicators (comparative fit index = 0.978, Tucker − Lewis index = 0.967, and root mean square error of approximation = 0.048 (95% CI 0.045-0.049)). As shown in Table 2, factor loadings were all statistically significant and ranged from a minimum of 0.543 to a maximum of 1.000. Meanwhile, the path coefficients between overall stem cell clinical trial quality and critical quality attributes were range from 0.760 to 0.953 with statistically significance.

### 3.4. Results of Quality Assessment of Stem Cell Trials

Among 37 trials, clinical significance of the research question was well presented and the average score was 0.791, followed by research design scored 0.581. Quality indicators scored low included implementation quality control strategy, product logistic and management plan, and participant protection, which were 0.189, 0.268, and 0.362, respectively (Table 3). Exploratory phase trials and confirmatory trials (phase 2/3 and phase 3) performed similarly in participant protection, scientific value, and quality control. Nevertheless, confirmatory trials had lower scores than early phase trials in material harvest (0.250 vs. 0.433) and stem cell products (0.300 vs. 0.406) at research sites.

## 4. Discussion

In this study, we proposed a new vision of proactive quality assessment tools of stem cell clinical trials facilitating by (1) reinforcing specific concerns of stem cell product, (2) understanding essential questions posed by stem cell clinical trial, (3) identifying the data and crucial activities in addressing them, and (4) protecting participants from severe event caused by stem cell treatment. To our knowledge, this study is the first reported attempt to develop a quality assessment tool for stem cell clinical trials. Results from structural equation modeling showed a good structural validity of the quality assessment tool.

As of highly innovation, complexity, and safety concerns, a comprehensive and feasible quality assessment tool is urgent as brand-new stem cell trials faced significant design and operational challenges. Similar to rapidly growing chimeric antigen receptor (CAR) T-cell therapy, stem cell trials went through a similar clinical development story: they are both in the early stage of clinical development, with smaller patient sample sizes and discrete distribution of median cell doses [21]. Improving design and conduct quantity of stem cell clinical research regulation remains a heat discussion across and within countries. Regulatory authorities issued guidelines to reiterate their stands of participant protection as well as their support for the development of stem cell-based therapy [22, 23]. Many countries support the acceleration of stem cell product clinical translation to satisfy unmet medical needs, whereas majority stem cell trials are in the early phase. A minimum guideline would leave intercenter variations on practice [24]. This phenomenon represents a significant implementation gap between regulatory goals and stem cell clinical trial design and conduct. Both researchers and research committees urge a systematic guideline to define key components of quality assurance, quality control, and participant protection in stem cell clinical trials.

We proposed a proactive quality assessment consideration for stem cell clinical trials of four aspects: *participant protection*, *scientific value*, *quality control*, and *stem cell products*. Proactive assessment is critical for conduct and quality improvements in clinical trials [25]. Traditionally, the quality of clinical trials has often been limited to two verification activities: quality control and quality assurance, which described in the ICH Good Clinical Practice Guidelines (ICH E6). As a consequence, the quality of clinical trials has been reactive for a long period. However, an approach emphasizing the prevention of error rather than correction should be the norm. The design of the protocol and the conduct of clinical trials should include an appropriate quality assessment process to make the study feasible and incorporate methods that help prevent important errors. Quality by design (QbD) has been used in the understanding of parameters in bioprocesses that allowed the robust production of stem cell products at manufacturers [26]. We therefore adopted QbD framework to identify key quality components of stem cell trials to enhance the capacity of self-regulate overall quality and participant protection.

Due to the distinct characters of living stem cell therapies, we emphasized the stem cell product management strategies in the development of quality assessment tool. Stem cell translation to clinical was usually recommended by *ISSCR Guidelines for Stem Cell Research and Clinical Translation* [27]. We developed a specific domain of quality indicators of stem cell products under consideration that not only does *ex vivo* processing of the stem cells (culturing, inducing, etc.) would affect cell's capacity to differentiate; risk of change in morphology, surface marker expression, and differentiation causing lower efficiency and higher risk of cell products may also occur during long-term storage in cryoprotectant agents, freezing, and thawing [28, 29]. Our study further included a comprehensive statement of producing parameters comparing to a few stem cell product storages, transportation, and disposal, which parts also regulated by GMPs in terms of ensuring the stability and sterilization of a stem cell dose [30, 31]. Take Cx601 (Alofisel) for example, adipose tissue derived, allogeneic mesenchymal stem cells, locally injected in perianal fistula tracts, is the first allogeneic stem cell therapy to be approved for the treatment of complex perianal fistulas in adult patients with Crohn's disease. The researchers clearly stated that Cx601 group received a single injection of 120 million Cx601 cells, which formulated in 24 mL of culture medium and shipped as four vials of 6 mL to the hospital and stored between 15°C and 25°C for a maximum of 48 h [32, 33]. Although QIs for cells produced by research institutes and manufacturers were measured differently, the principles were the same to ensure the same level efficiency and safety of cell products from freshly produced to bedside [34–36].

We further confirmed the structural validity of the QIs and critical quality attributes as latent variables. The SEM model revealed strong positive links from four quality attributes to the overall quality of a stem cell trial. The model also presented the importance of generating a good research question with a rigorous method, linking to high scientific value, and finally, high-quality trial. Stem cell product management strategy, measured jointly by cell harvest standards and SOPs, production standards and SOPs, and product logistic and management plan, has direct and indirect effects through general control strategy to the overall trial quality in our theory and confirmed by the model. Results of quality measurement were similar between exploratory and confirmatory trials among most QIs. No previous research was identified discussing the difference of trial quality by phases. We would reckon, for exploratory and confirmatory phase trials, the quality attributes were shared. However, the context might be justified differently based on mounts of previous knowledge and research purposes.

Our study has several limitations as well. First, the sample used to confirm the SEM was collected from peer-reviewed publications, which would be different in characteristics than in-planning studies. The quality and scientific value of published literature were recognized making the paper successfully went through the reviewing process; the “low-quality” studies were more likely to fail the process and could not be accessed. Second, the sample size was modest; therefore, the SEM may not be robust. Due to relatively few stem cell clinical trials that have been conducted to date, we failed to reach 200 samples for SEM [37]. CFI is a noncentrality parameter-based index, which designed to overcome the limitation of sample size effects [38]. We are expecting to further test the validity of processing part QIs with ongoing stem cell clinical trials. Third, in addition to the indicators listed in this study, stem cell clinical trials have more evaluation angles and quality indicators. It is well known that some parameters can be very critical to ensure the full or optimal exploration of clinical trials. Robustness of manufacturing processes of stem cells batches and beyond, such as stability, quality, logistics management from manufacturing to patients, and reproducibility of preparation before use in patients. Also, right formulations, dose, and time injection patterns of stem cell therapies were important quality indicators in stem cell trials. However, due to limited resource, we are here only for a proposal of a basic framework of quality assessment tool for stem cell clinical trials and failed to take all these details points into account. Improvement of quality assessment tool with more comprehensive and wider aspects of quality indicators is stilled warranted in the future research.

## 5. Conclusion

The results of critical quality attributes and quality indicators between expertise and confirmatory validation analysis are basically consistent, indicating the feasibility and validity of applying this quality assessment tool for overall quality evaluation of stem cell trials. The quality assessment tool for stem cell clinical trials showed here has enabled us to evaluate the overall quality of stem cell trials and therefore helps us refine quality practices in clinical stem cell trials. Future studies are needed with a focus on the reproducibility of stem cell measurement tool before strong recommendations can be made on its use.

## Figures and Tables

**Table 1:** Basic characteristics of included stem cell studies for validation of quality assessment tool.

Items of characteristics	Number of trials	Proportion (%)
Total	37	100.00
Trials status		
Complete or stop recruiting	32	86.49
Terminated	3	8.11
Unknown	2	5.41
Phase		
Phase 1	6	16.22
Phase 2	11	29.73
Phase 1/2	14	37.84
Phase 3	2	5.41
Phase 2/3	2	5.41
Unknown	2	5.41
Sponsor type		
Investigator-initiated trial	23	62.16
Industry-sponsored trial	14	37.84
Number of publications		
1	21	56.76
2~3	12	32.43
≥3	4	10.81
Cell type		
Pluripotent stem cells	3	8.11
Somatic stem cells	34	91.89

**Table 2:** Validation of quality assessment tool of stem cell clinical trials by structural equation modeling.

Quality attribute	Quality indicator	Estimate	Factor load	SE	*z* value	*P* value
Participant protection	Risk management and AE/SAE reporting plan	1.000	1.000			

Scientific value	Clinical significance of the research question	1.000	0.545			
	Research design	4.095	0.558	1.737	2.357	0.018^∗^

Quality control	Staffs/committees and definition of the roles	1.000	0.902			
	Data integrity	0.885	0.645	0.22	4.018	<0.001^∗^
	Implementation quality control strategy	1.904	0.759	0.398	4.781	<0.001^∗^

Stem cell products	Material harvest standards and SOPs	1.000	0.543			
	Stem cell production standards and SOPs	0.813	0.725	0.297	2.741	0.006^∗^
	Product logistic and management plan	0.854	0.676	0.32	2.67	0.008^∗^

Overall quality						
	Participant protection	1.000	0.760			
	Scientific value	0.172	0.953	0.064	2.716	0.007^∗^
	Control strategy	0.515	0.729	0.151	3.418	0.001^∗^
	Stem cell products	0.760	0.826	0.301	2.53	0.011^∗^

SE: standard error; SOP: standard operation procedure; ^∗^*P* value < 0.05.

**Table 3:** Results from quality assessment tool for stem cell clinical trials by phases of trials.

Quality attribute	Quality indicator	All (*n* = 37)		Exploratory phase^∗^ (*n* = 31)		Confirmatory phase^†^ (*n* = 4)	
		Mean	SE^§^	Mean	SE^§^	Mean	SE^§^
Participant protection	Risk management and AE/SAE reporting plan	0.362	0.185	0.373	0.190	0.322	0.178

Scientific value	Clinical significance of the research question	0.791	0.093	0.798	0.100	0.750	0.000
	Research design	0.581	0.187	0.581	0.198	0.563	0.161

Quality control	Staffs/committees and definition of the roles	0.372	0.249	0.383	0.255	0.359	0.240
	Data integrity	0.432	0.220	0.441	0.234	0.417	0.167
	Implementation quality control strategy	0.189	0.273	0.210	0.289	0.125	0.144

Stem cell products	Material harvest standards and SOPs	0.444	0.475	0.433	0.487	0.250	0.289
	Stem cell production standards and SOPs	0.389	0.290	0.406	0.303	0.300	0.258
	Product logistic and management plan	0.268	0.259	0.280	0.270	0.250	0.245

^∗^Exploratory phase includes phases 1, 1/2, and 2 trials. ^**†**^Confirmatory phase includes phase 2/3 and phase 3 trials. ^§^Standard error.

## Data Availability

The validation datasets used and/or stem cell clinical trial quality assessment tools are available from the corresponding author by request.

## Abbreviations

AE: Adverse event
CFI: Comparative fit index
COVID-19: Coronavirus disease 2019
GCP: Good Clinical Practice
GMP: Good Manufacturing Practice
ICH: International Conference on Harmonization
ISSCR: International Society of Stem Cell Research
PQRS: Progress-quality-regulation-scientific value
QbD: Quality by design
QI: Quality indicator
RMSEA: Root mean square error of approximation
SAE: Severe adverse event
SEM: Structural equation modeling
SOP: Standard operation procedure
TLI: Tucker-Lewis index.
